# Integrated DNA and RNA sequencing reveals targetable alterations in metastatic pediatric papillary thyroid carcinoma

**DOI:** 10.1002/pbc.28741

**Published:** 2020-10-03

**Authors:** Samara L. Potter, Jacquelyn Reuther, Raghu Chandramohan, Ilavarasi Gandhi, Faith Hollingsworth, Hadi Sayeed, Horatiu Voicu, Nipun Kakkar, Koel Sen Baksi, Stephen F. Sarabia, Monica E. Lopez, Daniel C. Chelius, Ioanna D. Athanassaki, Priya Mahajan, Rajkumar Venkatramani, Norma M. Quintanilla, Dolores H. Lopez-Terrada, Angshumoy Roy, D. Williams Parsons

**Affiliations:** 1 Texas Children’s Cancer Center, Department of Pediatrics, Baylor College of Medicine, Houston, Texas; 2 Department of Pathology and Immunology, Baylor College of Medicine, Houston, Texas; 3 Department of Pathology, Texas Children’s Hospital, Houston, Texas; 4 Department of Molecular and Human Genetics, Baylor College of Medicine, Houston, Texas; 5 Department of Surgery, Texas Children’s Hospital, Houston, Texas; 6 Michael E. DeBakey Department of Surgery, Baylor College of Medicine, Houston, Texas; 7 Department of Otolaryngology, Baylor College of Medicine, Houston, Texas; 8 Pediatric Endocrinology, Texas Children’s Hospital, Baylor College of Medicine, Houston, Texas; 9 The Dan L. Duncan Cancer Center, Baylor College of Medicine, Houston, Texas

**Keywords:** BRAF V600E, genomics, NTRK fusions, papillary thyroid carcinoma, pediatric thyroid cancer, RET fusions

## Abstract

**Background:**

Pediatric papillary thyroid carcinoma (PTC) is clinically and biologically distinct from adult PTC. We sequenced a cohort of clinically annotated pediatric PTC cases enriched for high-risk tumors to identify genetic alterations of relevance for diagnosis and therapy.

**Methods:**

Tumor DNA and RNA were extracted from FFPE tissue and subjected to next-generation sequencing (NGS) library preparation using a custom 124-gene hybridization capture panel and the 75-gene Archer Oncology Research Panel, respectively. NGS libraries were sequenced on an Illumina MiSeq.

**Results:**

Thirty-six pediatric PTC cases were analyzed. Metastases were frequently observed to cervical lymph nodes (29/36, 81%), with pulmonary metastases less commonly found (10/36, 28%). Relapsed or refractory disease occurred in 18 patients (18/36, 50%). DNA sequencing revealed targetable mutations in 8 of 31 tumors tested (26%), most commonly *BRAF* p.V600E (*n* = 6). RNA sequencing identified targetable fusions in 13 of 25 tumors tested (52%): *RET* (*n* = 8), *NTRK3* (*n* = 4), and *BRAF*. Mutually exclusive targetable alterations were discovered in 15 of the 20 tumors (75%) with both DNA and RNA analyzed. Fusion-positive PTC was associated with multifocal disease, higher tumor staging, and higher American Thyroid Association risk levels. Both *BRAF* V600E mutations and gene fusions were correlated with the presence of cervical metastases.

**Conclusions:**

Targetable alterations were identified in 75% of pediatric PTC cases with both DNA and RNA evaluated. Inclusion of RNA sequencing for detection of fusion genes is critical for evaluation of these tumors. Patients with fusion-positive tumors were more likely to have features of high-risk disease.

## 1 | INTRODUCTION

Papillary thyroid carcinoma (PTC) accounts for 90% of thyroid malignancies in children and is especially prevalent among females aged 15-19 years, in whom it is the second most common malignancy.^1^ Pediatric PTC patients are more likely to present with metastatic disease and have a higher likelihood of relapse as compared with their adult counterparts.^2,3^ Standard therapy for pediatric PTC most often involves total thyroidectomy (± lymph node dissection) as well as radioactive iodine therapy (RAI) for selected cases. Although the prognosis for patients who have undergone standard therapy is excellent, with a 15-year overall survival of 95%,^4^ there is significant morbidity secondary to surgical procedures and radiation exposure.^5^

Much of our knowledge regarding the genomic landscape of PTC can be attributed to The Cancer Genome Atlas (TCGA) project, which comprehensively analyzed 496 tumors and matched germline samples. This study revealed that adult PTC is characterized bya relatively quiet genome with mutually exclusive driving somatic genetic alterations in the mitogen-activated protein kinase (MAPK) and phosphatidylinositol 3-kinase (PI3K) pathways.^6^ However, as only five of the samples analyzed were from patients less than 19 years of age, the extent to which these findings are applicable to childhood PTC was not known. Instead, our understanding of the genomic landscape of pediatric PTC and corresponding clinical correlations relies on data generated from smaller pediatric cohorts analyzed by varying molecular methods.

These pediatric studies suggest that point mutations regularly found in adult PTC, such as *BRAF* V600E, are identified less frequently in children, whereas gene fusions are more common.^1,7–9^ Other studies note a correlation between the prevalence of oncogenic alterations and patient age, attributed to the more frequent presence of the *BRAF* V600E mutation in older patients.^10^ Unlike in adults, the presence of a *BRAF* V600E mutation in pediatric patients does not necessarily correlate with a more aggressive phenotype.^11,12^ Rather, recent papers have suggested that pediatric thyroid tumors driven by fusion genes, such as *RET/PTC1* and *NTRK3-ETV6*, may be correlated with more invasive disease.^13,14^

Analysis from the Chernobyl nuclear accident helped delineate some of the differences between radiation-induced and sporadic pediatric PTCs. Ricarte-Filho et al. found that radiation-induced pediatric PTCs have an increased prevalence of fusion oncogenes compared with sporadic tumors, with the vast majority of these fusions targeting genes (*RET*, *NTRK1* and *NTRK3*, *BRAF*) that activate the MAPK signaling pathway.^15^ *RET* rearrangements are also common in sporadic pediatric PTC, however, with rates ranging from 17% to 58%.^13–16^ Additionally, sporadic pediatric PTCs have been observed to have a larger number of point mutations compared with radiation-induced PTCs, particularly in *BRAF* and *RAS* genes.^15,17^

Although each of these studies has provided additional insight into the nature of pediatric PTC, the molecular methods utilized have varied significantly and largely relied on targeted analysis of known PTC genes. We sought to perform more extensive DNA- and RNA-based sequencing of a cohort of clinically annotated tumors, with a specific focus on patients with metastatic, relapsed, or refractory disease, in order to identify genetic alterations that might inform improved diagnostic and therapeutic approaches for these patients.

## 2 | MATERIALS AND METHODS

### 2.1 | Patients

**FIGURE 1:**
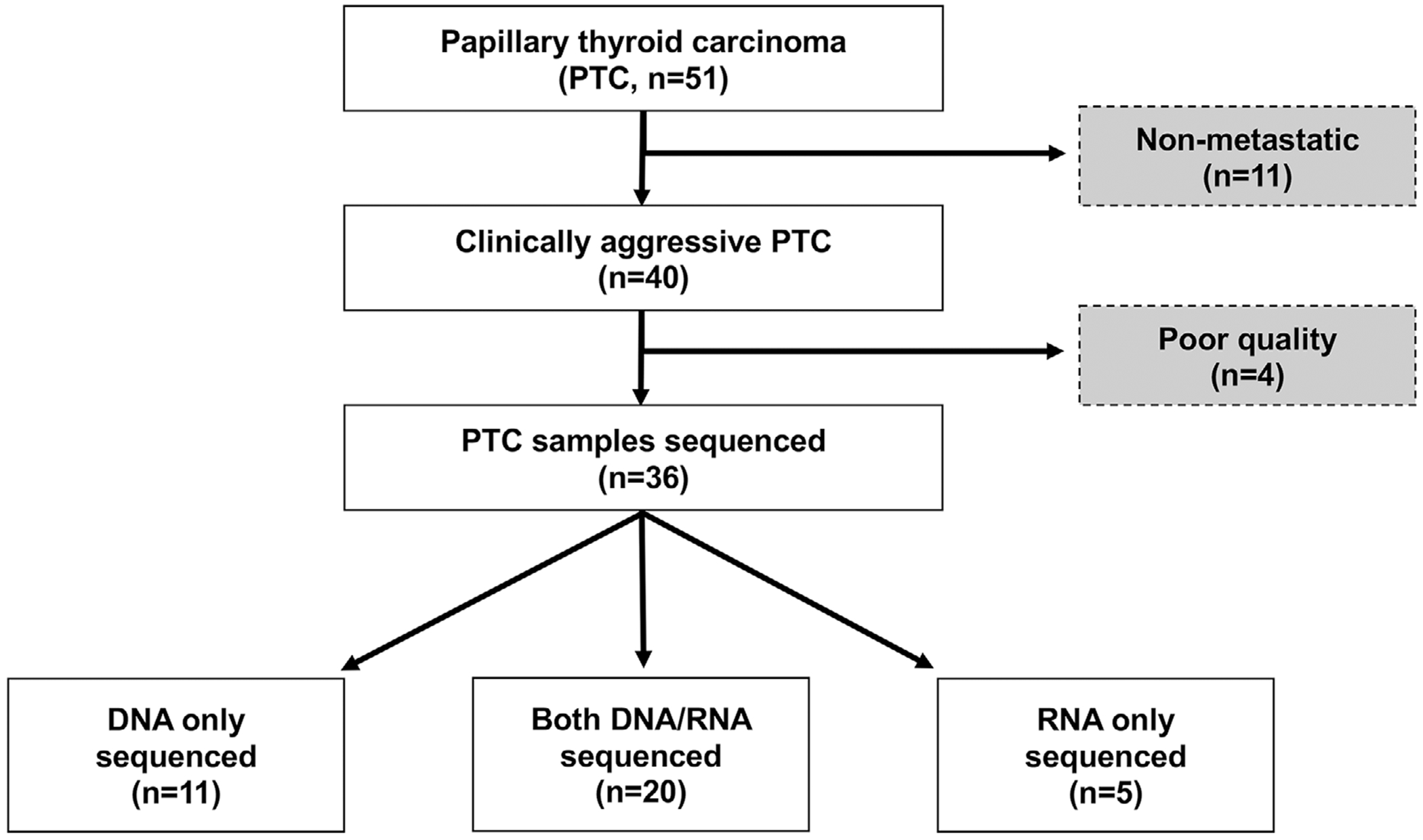
Flowchart demonstrating cohort selection and sequencing. PTC, papillary thyroid carcinoma

Potential cases of PTC were identified through review of the Texas Children’s Hospital pathology archives for the years 2005-2016, under a Baylor College of Medicine Institutional Review Board–approved protocol for clinical and genomic analysis of rare tumors. Patients 0-24 years of age were considered for inclusion. A total of51 PTC cases were identified for clinical review, with metastatic, relapsed, and treatmentrefractory cases prioritized for analysis (Figure 1). Chart review was performed by accessing information available in the electronic medical record, including radiology and pathology reports, laboratory results, and clinical notes from oncology, endocrinology, and surgical specialties. References to potential cancer susceptibility testing were noted and related records were reviewed if relevant. Clinical characteristics were annotated by pediatric oncologists who regularly participate in the Thyroid Tumor Program at Texas Children’s Hospital (SLP, PM, and RV). Ultimately, 40 cases were selected, all of which were diagnostic (pretreatment) specimens obtained from either lobectomy or total thyroidectomy. Of note, 14 of the patients included in this cohort were also included in a prior study that included more targeted gene testing (Supporting Information Table S1).^18^ Disease status was classified using the American Joint Committee on Cancer TNM classification system for differentiated thyroid carcinoma, 7th edition, and risk level was assessed as outlined in the American Thyroid Association (ATA) management guidelines for children with differentiated thyroid cancer.^19^

### 2.2 | Tumor sequencing

A pathologist with specific expertise in thyroid tumors (NMQ) examined the hematoxylin and eosin–stained tumor slides to identify representative areas of tumor suitable for molecular testing. DNA and RNA were extracted from FFPE tumor specimens according to the manufacturers’ protocols utilizing the QIAamp DNA FFPE Tissue Kit (Qiagen) and the RecoverAll Total Nucleic Acid Isolation Kit for FFPE (Ambion), respectively. DNA and RNA were quantified by Qubit (Thermo Fisher Scientific), with RNA undergoing an additional quality assessment by the quantitative PCR assay PreSeq (ArcherDx).

Of the original 40 cases selected for sequencing, four cases were excluded due to insufficient quantity and/or quality of nucleic acids. Therefore, 36 tumor samples with adequate quantity and quality of nucleic acids for sequencing were utilized for NGS library preparation. DNA NGS libraries were generated using a custom hybridization capture panel (NimbleGen/Roche) designed to capture all coding exons of 124 genes associated with pediatric solid tumors as well as the *TERT* gene promoter.^20^ RNA NGS libraries were generated using the Oncology Research Panel (ArcherDx) that utilizes Anchored Multiplex PCR chemistry to capture gene fusion events involving 75 recurrently rearranged cancer genes.^21^ NGS libraries were sequenced 4- to 6-plex on an Illumina MiSeq using V2 and V3 chemistry, respectively, and demulitplexed using bcl2fastq v2.18.0.12 (Illumina), achieving approximately 2.2-4.4 million sequencing reads per sample. The resulting DNA FASTQ files were analyzed using a custom DNA bioinformatics pipeline that includes alignment by BWA v0.7.12^22^ (Wellcome Trust Sanger Institute) and deduplication and QC analysis by Picard v2.9.2.^23^ Variant calling was completed by NextGENev2.4.1.2 (SoftGenetics) and Platypus v0.8.1^24^ (Wellcome Trust Centre for Human Genetics) for the detection of SNVs and short indels, with variant annotation by VEP v.89^25^ (Ensembl). Copy-number analysis was performed using CNVkitv0.9.3.^26^ CNVkit performs circular binary segmentation on the normalized GC and target capture density bias corrected log2 difference of binned read depths, using on-target (average bin size = 300 bp) and off-target reads (average bin size = 500 000 bp), between the tumor sample and reference pooled-normal peripheral blood samples to identify genome-wide regions of CNVs. Gene-level deletions and amplifications were then derived using these copy-number segments. RNA FASTQ files were analyzed by the Archer Analysis suite v4.1.0.6 (ArcherDx) with additional custom wrapper and text-processing scripts for the detection of gene fusion events.

### 2.3 | Statistical analysis

Fisher exact test was performed to test for the association of tumor alterations with specific clinical features. Samples were included in this analysis if a driver alteration, defined as a *BRAF* V600E mutation or an oncogenic gene fusion, was identified. Samples sequenced using only one methodology (DNA testing or RNA testing) and without an identified driver alteration were not considered completely analyzed and therefore not included in this analysis.

## 3 | RESULTS

### 3.1 | Demographic and clinical characteristics

Our cohort consisted of 36 patients with pediatric PTC. Of those, 34 were diagnosed between ages 8 and 19 years, with the majority of patients being 15-19 years (Table 1). The male-to-female ratio was 1:3. Metastasis to cervical lymph nodes occurred in 29 of the 36 patients (81%), of which 10 also had lung metastases (28%). Tumor relapse postsurgery occurred in 18 of the patients (50%). In addition, although the majority of our cohort was comprised of patients without prior history of irradiation, we did include eight patients (22%) who had undergone radiation therapy, including two patients with a history of Hodgkin lymphoma and four patients who received whole body irradiation prior to bone marrow transplant (two with acute myelogenous leukemia, one with chronic myelogenous leukemia, and one with Hurler syndrome). The two remaining patients who had received radiation had previous diagnoses of Wilms’ tumor and acute lymphoblastic lymphoma. The time from prior radiation to diagnosis of thyroid cancer ranged from 6 to 18.5 years (median 10.9 years). One patient was diagnosed with Li-Fraumeni syndrome after her PTC diagnosis; no other patients had positive germline testing results indicating known cancer predisposition syndromes. All patients underwent surgical resection of the thyroid gland, and 34 of 36 received RAI (94%). Tumors ranged in size between 0.2 and 7 cm, with a median size of 2.2 cm.

### 3.2 | DNA mutation panel testing

**FIGURE 2:**
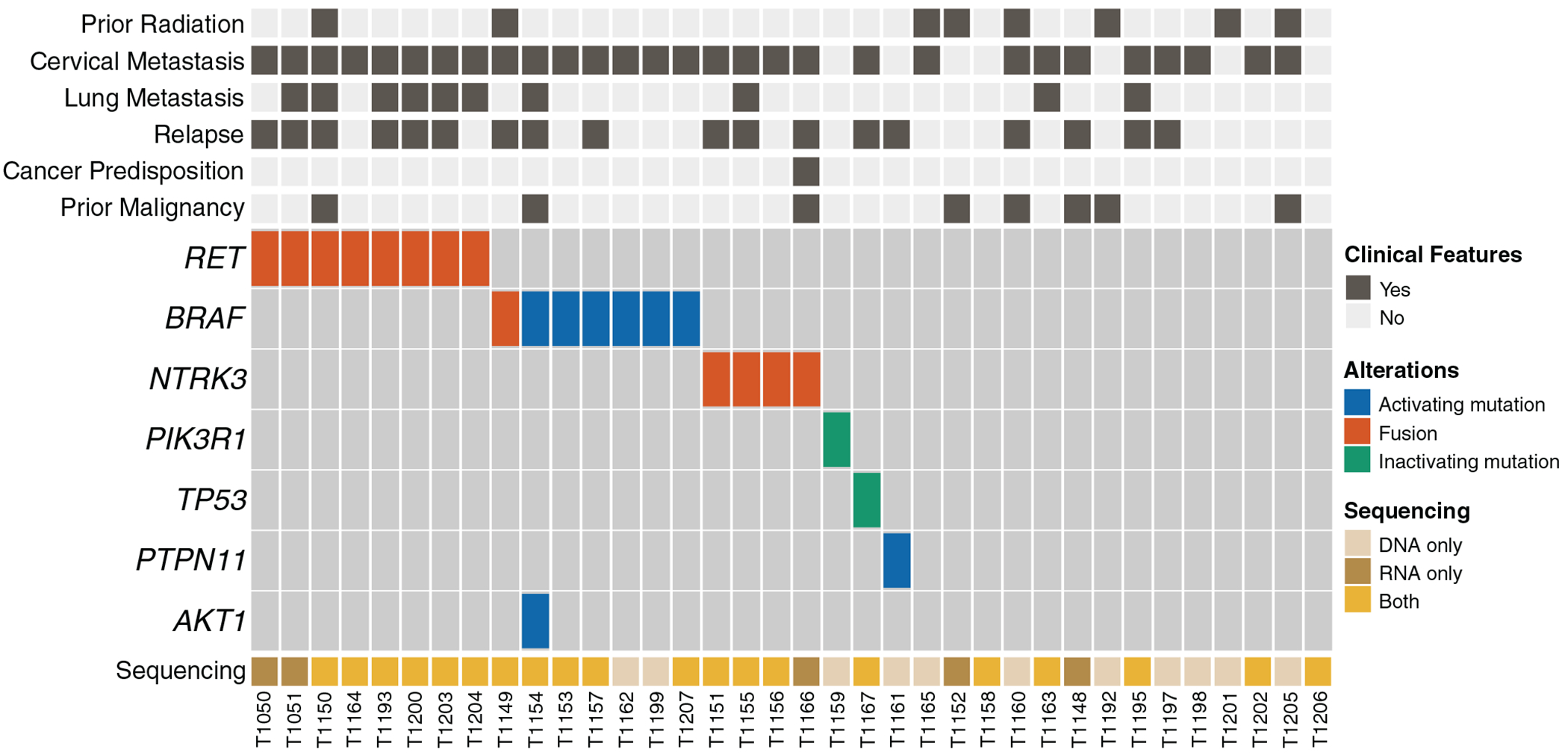
Tumor sequencing findings and selected clinical characteristics

DNA sequencing for mutation and copy-number profiling was successfully completed for 31 cases (Figure 1), revealing 10 cancer gene mutations in nine tumor samples (29%; Figure 2). The most common mutation identified was *BRAF* p.V600E, found in six tumors (19%) including one tumor with a co-occurring *AKT1* p.E17K mutation, a known hotspot driver mutation in the PI3K pathway that has been identified in multiple cancer types, including in PTC.^27–29^ Other alterations identified included a *PTPN11* hotspot mutation (p.S502T), a frameshift mutation in *PIK3R1* (p.Y29fs), and a nonsense mutation in *TP53* (p.Y163X). The genome-wide copy-number profiles for these tumor samples did not reveal high-level amplifications or homozygous losses (Supporting Information Figure S1).

### 3.3 | RNA fusion panel testing

**FIGURE 3:**
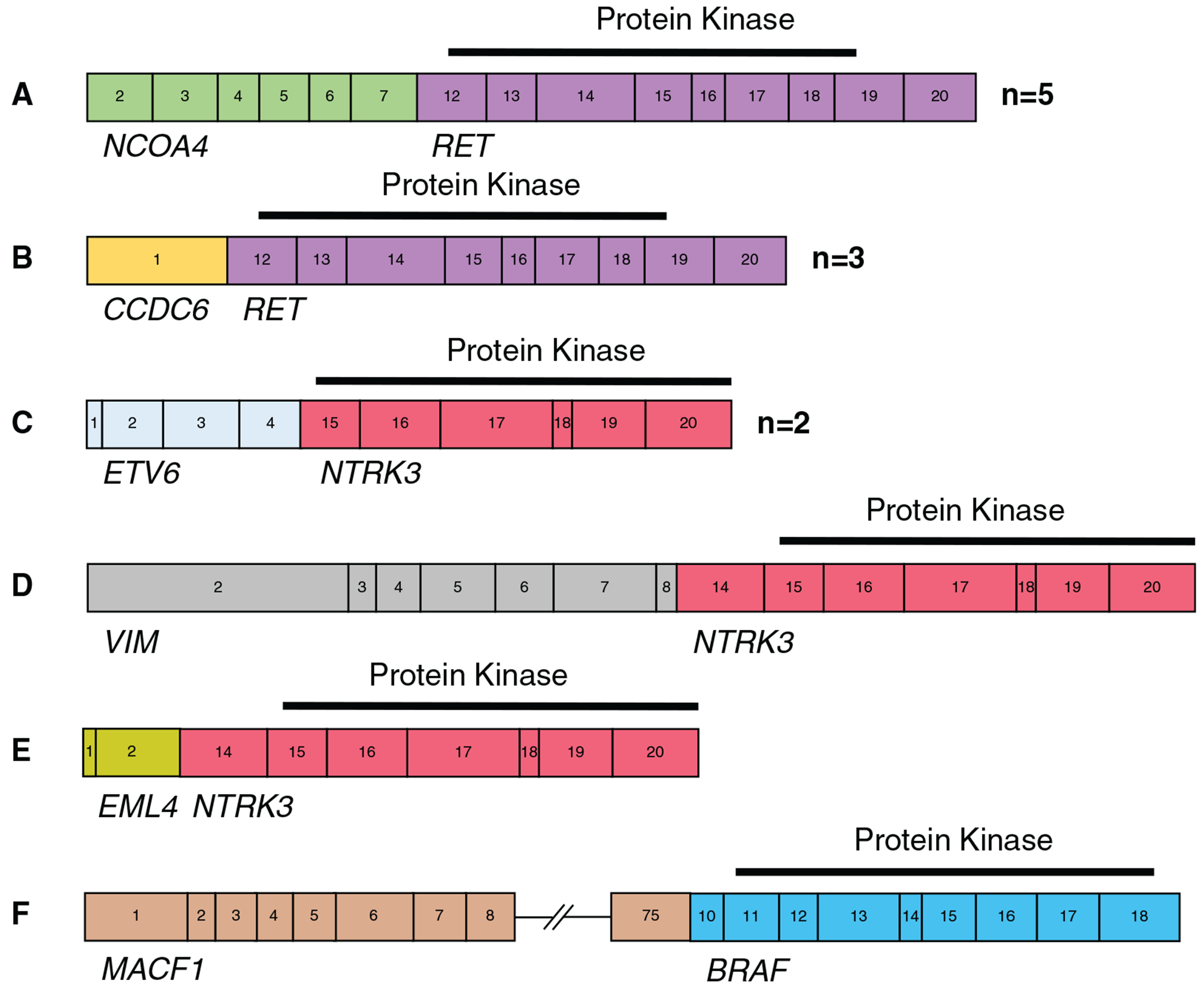
Fusions detected by RNA panel sequencing: (A) *NCOA4-RET*, (B) *CCDC6-RET*, (C) *ETV6-NTRK3*, (D) *VIM-NTRK3*, (E) *EML4-NTRK3*, and (F) *MACF1-BRAF*. All fusions retain the protein kinase domain

RNA fusion panel testing was successfully completed for 25 cases (Figure 1), revealing fusions in 13 tumor samples (52%; Figure 2). The majority of these (*n* = 8) were activating *RET* kinase fusions, including the well-known *NCOA4-RET* fusions (also known as *RET/PTC3*) in five cases and *CCDC6-RET* (also known as *RET/PTC1*) in three tumors (Figure 3). Four other tumors harbored *NTRK3* fusions, including two *ETV6-NTRK3* fusions, one *EML4-NTRK3* fusion, and a novel *VIM-NTRK3* fusion in one sample. Finally, one tumor was found to harbor a *MACF1-BRAF* fusion. All fusion genes identified retained the kinase domains of the oncogenic partners (*RET*, *NTRK3*, and *BRAF*).

### 3.4 | Clinical associations

**FIGURE 4:**
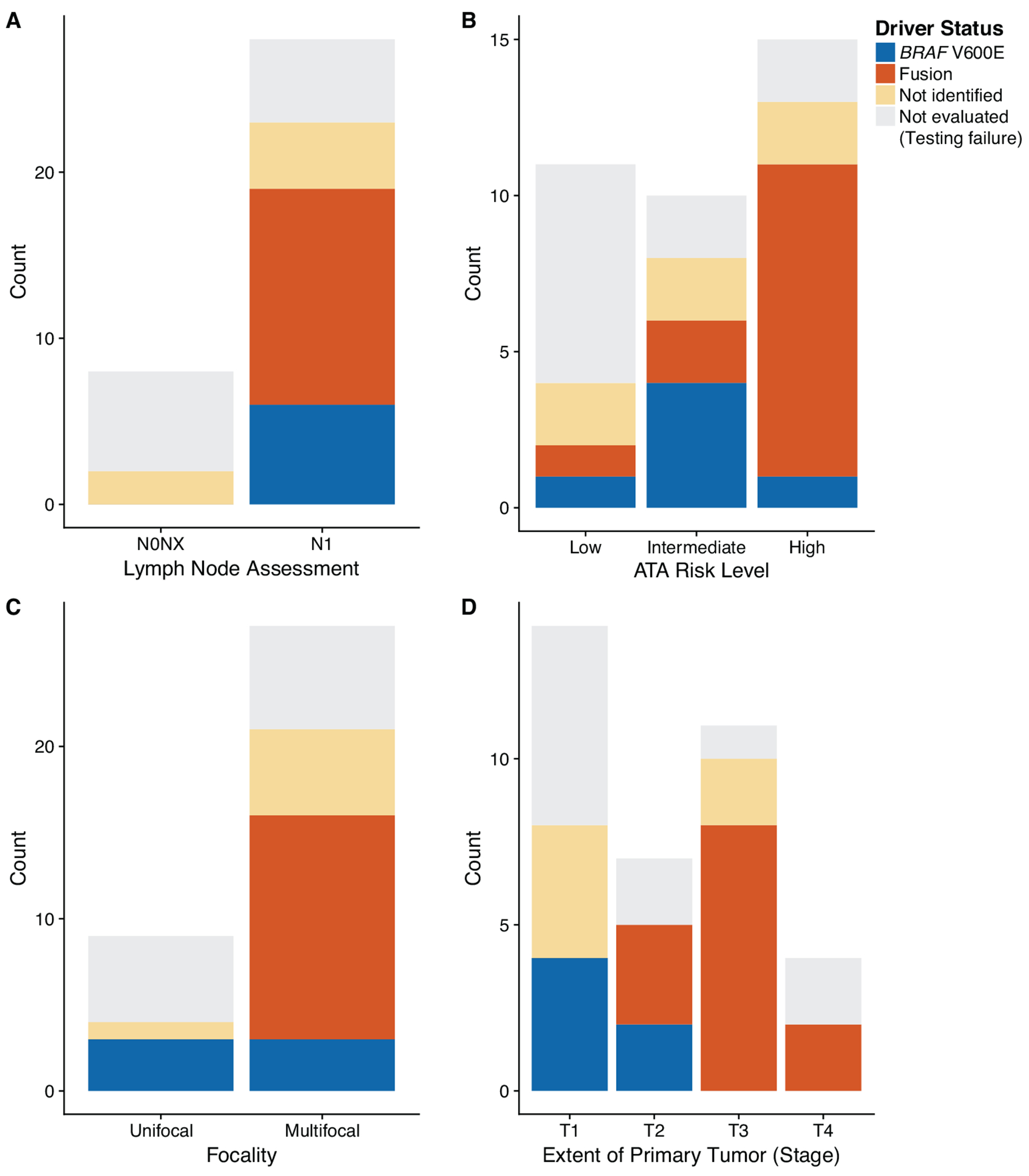
Distribution of driver alterations (gene fusions and *BRAF* V600E mutations) with various clinical characteristics, including (A) cervical lymph node metastases, (B) ATA risk level, (C) primary tumor focality, and (D) extent of primary tumor

Driver alterations were found to be associated with cervical metastases or N1 classified lymph node samples (*P* = 0.05, 19/23; Figure 4a). The presence of an activating kinase gene fusion correlated with higher ATA risk level (*P* = 0.027, 10/13 high-risk patients; Figure 4b) and multifocal disease pathology (*P* = 0.039, 13/22 multifocal tumors; Figure 4c). *BRAF V600E* mutations were more frequent in smaller tumors (T1 and T2), whereas fusions were more frequent in larger tumors, T2 and higher (*P* = 0.001; Figure 4d).

## 4 | DISCUSSION

Integrated DNA/RNA panel sequencing successfully identified frequent clinically relevant alterations in a cohort of pediatric PTC patients enriched for metastatic and relapsed/refractory disease. Overall, *BRAF* V600E mutations were present in 19% of samples (6/31), consistent with other recent pediatric studies that have reported frequencies ranging from 17% to 61%.^1,11,14,17,30,31^ In contrast with adult PTC, fusions were detected more than twice as frequently as *BRAF* V600E mutations in our cohort, with activating kinase fusions detected in 52%of samples tested (13/25). All but one of the driver alterations detected in this cohort were mutually exclusive (a single driver alteration per patient). This is consistent with our understanding of adult PTC development, in that these tumors usually arise from a single molecular driver whose alteration causes continuous activation of the MAPK and PI3K signaling pathways.^6,7^

Fusion-positive patients were more likely to be classified as ATA high-risk status and to have multifocal disease, findings that hold implications for future molecular testing. These patients are more likely to develop relapsed disease requiring repeat surgery or RAI treatments. In comparison, patients with *BRAF* V600E mutations often presented with smaller primary tumors (T1 and T2) than those with fusion-positive disease, although they frequently were found to have cervical lymph node metastasis at the time of diagnosis.

Employing the partner-agnostic Oncology Research Panel (ArcherDx) enabled us to identify multiple unexpected fusions in our cohort. For example, three tumors were found to harbor fusions not known to be previously reported in PTC, including the patient with a novel *VIM-NTRK3* fusion. A second patient was discovered to have PTC containing an *EML4-NTRK3* fusion. This fusion has been identified in patients with infantile fibrosarcoma,^32,33^ but not previously in PTC. Interestingly, this patient had a prior history of relapsed osteosarcoma and later developed malignant melanoma, and was found to have Li-Fraumeni syndrome by targeted *TP53* clinical testing; this was not detected in our analysis as the DNA panel testing was not successful. Finally, a *MACF1-BRAF* fusion was identified in a third patient who had previously been diagnosed with AML and received radiation prior to bone marrow transplant; this fusion is novel in PTC and has been rarely reported in low-grade glioma.^34^

Our cohort contained a number of individual patients with notable genomic and clinical findings. For instance, a patient with a history of short stature and delayed puberty was found to have tumor containing a *PTPN11* hotspot mutation (p.S502T) with a variant allele frequency of 50%. Although a blood sample was not available for germline testing, this mutation has previously been detected in patients with Noonan’s syndrome.^35^ Another patient—the only case with two driver alterations detected—was found to have *BRAF* and *AKT1* mutations at different variant allele frequencies (37% and 22%, respectively) in their tumor sample, suggesting that the *AKT1* mutation was present in a subclonal population of the tumor.

Importantly, the vast majority of the alterations identified in our cohort are currently targetable with FDA-approved or investigational agents, including MAPK pathway inhibitors, PI3K pathway inhibitors, and other kinase inhibitors. Vemurafenib, a potent BRAF inhibitor that is specific for tumors with the *BRAF* V600E mutation, has shown antitumor efficacy in adults with progressive metastatic, RAI-refractory *BRAF* V600E-positive PTC.^36,37^ Although its use in pediatric patients has generally been limited, in part due to the difficulty of studying adequate numbers of pediatric PTC patients in clinical trials,^38^ there are a number of case reports of children with *BRAF* V600E-positive tumors whose disease responded to treatment.^39–41^

One recently FDA-approved agent is larotrectinib, a highly selective small-molecule inhibitor of the tropomyosin receptor kinase (TRK) proteins (encoded by kinase genes *NTRK1*, *NTRK2*, and *NTRK3*), which has demonstrated potent antitumor efficacy in both children and adults with TRK fusion-positive tumors.^42,43^ Notably, TRK fusion-positive tumors comprised approximately 11% (4/36) of our cohort. Results from a phase I trial included two pediatric patients with TRK (*NTRK1* and *NTRK3*) fusion-positive PTC; although these two patients could not be objectively evaluated by RECIST criteria (as they did not have measurable disease at enrollment), both patients remained on treatment without progression at the data cutoff point over seven months later.^43^

Multiple targeted agents have been developed for adult patients with RET driven solid tumors. Sorafenib and lenvatinib, multikinase inhibitors that target *RET*, *FLT1, KDR, FLT4*, *PDGFRA, PDGFRB*, and *KIT*, are FDA approved for adults with PTC and *RET* fusions.^44^ Sorafenib has been studied in a phase 2 trial in pediatrics but no PTC patients were enrolled on study.^45^ A recent report of three pediatric patients with refractory PTC who demonstrated clinical improvement with lenvatinib suggests that this may also be of potential utility in relapsed or refractory patients.^46^ Additionally, selpercatinib, an oral and selective investigational drug targeting RET kinase abnormalities, was recently FDA approved for patients ages 12 and older with metastatic or advanced *RET*-mutated medullary thyroid carcinoma or *RET* fusion–positive (and RAI-refractory) thyroid cancer.^47^ Selpercatinib will soon be available to relapsed pediatric patients through the National Cancer Institute and Children’s Oncology Group jointly sponsored Pediatric MATCH trial.^48^

Of the 20 cases that were evaluated by both DNA and RNA NGS panels, potentially clinically relevant alterations were detected in 15 cases (75%). The frequency of driver alterations in metastatic and relapsed/refractory pediatric PTC, in combination with a steady increase in available molecularly targeted agents and the need to decrease morbidity from repeated surgeries and RAI treatments, has significant implications for the utility of molecular testing in this patient population. Our findings strongly support the inclusion of RNA testing in such analysis, especially in ATA high-risk patients where the diagnostic yield is particularly high. Given the potential therapeutic importance of identifying targetable gene fusions that are often characterized by diverse and novel gene partners,^49^ methods that enable partner-agnostic detection of fusion genes, such as anchored multiplex chemistry (used in this study to detect a novel *VIM-NTRK3* fusion) or capture-based transcriptome sequencing, should be preferred. At our center, we clinically test all relapsed and/or refractory pediatric patients with PTC using paired targeted DNA/RNA cancer gene panels as described above, and recommend upfront tumor testing in patients who are not amenable to conventional management, including RAI therapy, or who have symptomatic lung disease; however, a stepwise approach, in which such patients are first evaluated for *BRAF* V600E mutations, and if negative, undergo fusion testing, isa reasonable alternative. Migration of targeted agents from salvage regimens toward frontline treatment remains challenging; however, as both physicians and patients gain experience with these therapies in pediatrics, we anticipate the demand for such treatments will increase for selected patients.

Our retrospective study was constrained bya relatively small cohort size, which limited our ability to draw definitive conclusions when clinically correlating our results. Additionally, as a retrospective study, we were limited in terms of quality and amount of available samples for sequencing, as molecular testing had not been prioritized at the time these specimens were obtained.

In conclusion, our experience suggests that targeted DNA mutation and RNA fusion panel sequencing for pediatric patients with ATA high-risk PTC has the potential to be of clinical benefit, especially with the recent increase in available targeted agents for pediatric patients. We anticipate that as we continue to attempt to minimize morbidity associated with repeated RAI exposure and surgery for these patients, utilization of molecularly targeted agents in conjunction with current standard therapies will increase, particularly among patients with lung metastases and refractory disease. Additionally, as our cohort consisted of pretreatment specimens obtained from thyroidectomy, further studies evaluating the degree of tumor evolution over time and necessity for rebiopsy will be needed.

## Supplementary Material

Supporting Information Figure S1

Supporting Information Table S1

Additional supporting information may be found online in the Supporting Information section at the end of the article.

## Figures and Tables

**TABLE 1:** Demographic and clinical characteristics of the pediatric PTC cohort

Clinical characteristics		
	Number (*n* = 36)	Percent
Sex		
Male	9	25
Female	27	75

Age		
<10	2	6
10-14	10	28
15-19	22	61
20-23	2	6

Metastasis	29	81
Cervical lymph node	29	81
Lung	10	28
Relapse/progression	18	50

Prior irradiation	8	22

PTC, papillary thyroid carcinoma.

## Data Availability

The data that support the findings of this study are available on request from the corresponding author. The data are not publicly available due to privacy or ethical restrictions.

## ABBREVIATIONS:

ATA: American Thyroid Association
DNA: deoxyribonucleic acid
FFPE: formalin-fixed paraffin-embedded
MAPK: mitogen-activated protein kinase
NGS: next-generation sequencing
PI3K: phosphatidylinositol 3-kinase
PTC: papillary thyroid carcinoma
RAI: radioactive iodine
RNA: ribonucleic acid
TCGA: The Cancer Genome Atlas
TRK: tropomysin receptor kinase
XRT: radiation therapy
